# Role of Membrane Cholesterol Levels in Activation of Lyn upon Cell Detachment

**DOI:** 10.3390/ijms19061811

**Published:** 2018-06-19

**Authors:** Takao Morinaga, Noritaka Yamaguchi, Yuji Nakayama, Masatoshi Tagawa, Naoto Yamaguchi

**Affiliations:** 1 Division of Pathology and Cell Therapy, Chiba Cancer Center Research Institute, Chiba 260-8717, Japan; mtagawa@chiba-cc.jp; 2 Laboratory of Molecular Cell Biology, Graduate School of Pharmaceutical Sciences, Chiba University, Chiba 260-8675, Japan; yamaguchinoritaka@chiba-u.jp (N.Y.); nyama@faculty.chiba-u.jp (N.Y.); 3 Department of Biochemistry and Molecular Biology, Kyoto Pharmaceutical University, Kyoto 607-8414, Japan; nakayama@mb.kyoto-phu.ac.jp; 4 Department of Molecular Biology and Oncology, Graduate School of Medicine, Chiba University, Chiba 260-8670, Japan; mtagawa@chiba-cc.jp

**Keywords:** cholesterol, Src-family kinases, subcellular localization, membrane distribution, Lyn activation, cell–scaffold interactions, cell detachment

## Abstract

Cholesterol, a major component of the plasma membrane, determines the physical properties of biological membranes and plays a critical role in the assembly of membrane microdomains. Enrichment or deprivation of membrane cholesterol affects the activities of many signaling molecules at the plasma membrane. Cell detachment changes the structure of the plasma membrane and influences the localizations of lipids, including cholesterol. Recent studies showed that cell detachment changes the activities of a variety of signaling molecules. We previously reported that the localization and the function of the Src-family kinase Lyn are critically regulated by its membrane anchorage through lipid modifications. More recently, we found that the localization and the activity of Lyn were changed upon cell detachment, although the manners of which vary between cell types. In this review, we highlight the changes in the localization of Lyn and a role of cholesterol in the regulation of Lyn’s activation following cell detachment.

## 1. Introduction

Cholesterol is a precursor for bile acid, steroid hormones, and vitamin D, and is a major component of cellular membranes [1]. Marked elevation of serum cholesterol is believed to be a cause of various diseases, although the association between disease mortality and serum cholesterol levels remains controversial [2,3]. Cholesterol is considered to be unevenly distributed in various cellular membranes, forming cholesterol-rich domains within membranes termed as lipid rafts, and may affect the activities of various types of membrane proteins or membrane-anchored proteins [4]. Influence of changes in cellular cholesterol levels and cholesterol distribution among cellular membranes on cell signaling is still under investigation.

Src-family kinases—including c-Src, Lyn, Fyn, c-Yes c-Fgr, Hck, Lck, and Blk—are membrane-anchored tyrosine kinases localized on the cytoplasmic side of the cell membrane and are important regulators of signal transduction, thereby playing roles in cellular processes, such as cell adhesion, tumor malignancy (including apoptosis resistance, cell growth, and transformation), and immune signals [5,6,7,8,9]. The N-terminal Src homology (SH) 4 domain of Src-family kinases has a glycine residue that undergoes a post-translational modification with myristic acid, and some have one or two cysteine residues that can be modified with palmitic acid [10]. These lipid modifications serve to anchor the Src-family kinases onto cellular membranes and affect their trafficking and localizations [11]. In addition, Src-family kinases commonly possess SH3 and SH2 domains, which mediate protein–protein interactions, and a C-terminal kinase domain [12]. According to microscopic and biochemical analyses of their distributions within cellular membranes, Src-family kinases can be distributed in both lipid rafts and non-raft membranes, in which their activities are differentially regulated [13,14].

Cell adhesion, including cell attachment to the extracellular matrix and cell–cell contact between adjacent cells, recruits various signaling molecules to the plasma membrane [15]. Responses of signaling pathways of the cell adhesion apparatus vary between cell types because of the differences in the function and the stiffness of each tissue where those cells engraft [15,16]. For example, cell–scaffold interactions are essential for epithelial cell survival, and their loss triggers apoptotic cell death termed anoikis [17]. Formation of cell polarity in monolayered epithelial cells requires cell–cell adhesion, such as adherens junction [5]. Meanwhile, leukocytes can survive in the blood stream without cell–scaffold interactions but require cell–cell adhesion for migration to sites of injury [18]. Activation of Src-family kinases following cell adhesion is involved in many downstream signaling pathways, from stabilization of adherens junctions in epithelial cells to rolling and spreading of leukocytes on endothelial cells [5,18]. Interestingly, dissociation of cell–scaffold interactions also activates Src-family kinases and activates signal transduction pathways, such as anoikis resistance in suspended cells [17].

Cell detachment can change the localizations of many molecules associated with the plasma membrane, including cholesterol and Src-family kinases. In this review, we will focus on the regulation of the activities of Src-family kinases following cell detachment, based on our recent results that show a strong relationship between the distribution of membrane cholesterol and the localizations of Src-family kinases.

## 2. Membrane Cholesterol Affects the Activities of Signaling Molecules

### 2.1. Heterogenous Cholesterol Distribution among Cellular Membranes

Cholesterol provided by internal synthesis through the mevalonate pathway and external delivery by lipoproteins accounts for around 20 mol % of cellular lipids [19]. The expression levels of enzymes and receptors working in these pathways are precisely regulated to maintain the total cholesterol level in each cell [20]. Cholesterol is predominantly distributed to the plasma membrane; some is also distributed to the Golgi apparatus and endosomes; alternatively, the endoplasmic reticulum (ER) contains very little cholesterol (<5 mol %) [1,21,22], despite cholesterol being synthesized in the ER through the mevalonate pathway. This heterogeneous distribution of cholesterol occurs through vesicular and non-vesicular transport. Caveolin, for instance, directly associates with cholesterol and forms transport vesicles [23]. Non-vesicular routes for cholesterol transport involve various lipid-transfer proteins, such as steroidogenic acute regulatory-related lipid-transfer proteins, oxysterol-binding homology proteins, and Niemann–Pick disease type C (NPC) proteins [22,24]. In the external delivery pathway, low-density lipoproteins (LDL) are first captured by the receptors on the cell surface, and are then transported to late endosomes/lysosomes by clathrin-coated vesicles. Free cholesterol is generated in late endosomes/lysosomes from esterified cholesterol derived from LDL, and is transferred from the endosomal lumen to the membrane by the intracellular cholesterol transporters NPC-1 and NPC-2 [25]. Impaired intracellular trafficking of cholesterol can be a cause of disease; NPC, which is caused by mutations in *NPC1* or *NPC2*, cause excessive accumulation of unesterified cholesterol in late endosomes/lysosomes [26]. Reasons for the heterogeneous distribution of cholesterol within cellular membranes are still unclear. Given the hypothesis that cellular membranes are largely classified by their components into two territories, the plasma membrane—trans-Golgi—endosome territory and the ER—nuclear envelope—*cis*-Golgi territory, cholesterol is primarily distributed in the former territory [27].

In addition to the varied cholesterol distribution among cellular compartments, lateral distribution of cholesterol within a membrane is also considered heterogeneous. Membrane cholesterol aggregates and recruits other saturated lipids, which may form lipid rafts. Lipid rafts, which recruit specific proteins and serve as specialized signaling platforms, are considered to be dynamically generated 10–200-nm-diameter structures in the plasma membrane [3]. Studies investigating the composition and functional role of lipid rafts are currently under progress. For example, numerous studies have isolated detergent-insoluble membrane proteins using non-ionic detergent-based fractionations, and have examined the relationship between the activities and the distributions of the membrane proteins. Many other methods, including the analysis of microscale phase separation in giant plasma membrane vesicles, have been tested. However, the raft hypothesis could not be sufficiently demonstrated using these methods because of the possibilities that these techniques themselves may cause artificial effects on biological membranes [28]. A commonly used non-ionic detergent (e.g., Triton X-100) can interfere with the formation of membrane domains [29], suggesting that membrane fractionations using detergents may provide artificial results.

### 2.2. Influence of Changes in the Level of Membrane Cholesterol on the Signaling Molecules

Although the raft hypothesis is challenged by the latest approaches, several lines of evidence have demonstrated the importance of cholesterol for signal transductions at the cellular membrane. A lipidomic analysis of lipid rafts formed at membrane segments harboring activated T-cell receptors revealed that the cholesterol concentration of the lipid rafts was approximately 50 mol %, whereas that of the whole cell membrane was approximately 20 mol % [30]. Given that the maximum solubility of cholesterol in lipid bilayers comprising phosphatidylcholine or phosphatidylethanolamine is 66 mol % or 51 mol %, respectively [31], we have assumed that lipid rafts may constitute cholesterol-saturated sections of the membrane. Many studies using a cholesterol remover, methyl-β-cyclodextrin (MβCD), and water-soluble cholesterol revealed that the kinase activity of the epidermal growth factor receptor (EGFR) is upregulated by cholesterol depletion, and downregulated by cholesterol incorporation [32,33]. However, the downstream effects of EGFR kinase activity induced by cholesterol depletion were slightly different from those induced by EGF binding. The activity of Akt, for example, was inactivated by cholesterol depletion and activated by cholesterol incorporation [34].

Cholesterol depletion increases Src-dependent phosphorylation of EGFR tyrosine 845 [32], indicating that Src-family kinases are involved in the activation of EGFR signaling induced by cholesterol depletion. Although Src-family kinases and EGFR reciprocally activate each other [35,36], Src-family kinases are, in some cases, not significantly activated or inactivated by cholesterol depletion [37,38]. In addition, active Src increases at focal contact sites under cholesterol-depleted conditions [37]. Moreover, cholesterol depletion activates the Src-family kinases in cells expressing shRNA against desmoglein-2, a component of cell–cell adhesion [39]. Therefore, we hypothesize that the activities of Src-family kinases under cholesterol depletion is influenced by cell adhesion.

## 3. Cell–Scaffold Interaction Affects the Activities of Src-Family Kinases

### 3.1. Involvement of Cell Adhesion in Cell Functions

Various signals related to cellular functions are regulated by the surrounding microenvironment. This includes the expression of tissue-specific genes, such as *β-casein* gene expression in mammary cells which responds to extracellular matrix (ECM) due to the ECM-response element in the promoter region [40]. Multi-drug resistance in cancer cells is affected by changes in cell–scaffold interactions [16]. Even tumorigenesis in cells expressing the transforming protein v-Src was attenuated by the environmental conditions in chicken embryo wings [41]. Cell surface interacts with the external fluid, adjacent cells, and the extracellular matrix. Similar to the signals activated by growth factors or chemokines in the external fluid, the signals from cell–cell and cell–scaffold interactions affect cell functions, proliferation, and mobility [15]. These interactions organize the polarized molecular trafficking pathways, and induce formation of the specific membrane domains essential for cellular functions, such as the apical and basal membranes in monolayered epithelial cells [42] and the immunological synapses in lymphocytes [43].

### 3.2. Influences of Cell Detachment on the Activities of Src Family Kinases

Loss of cell–scaffold interactions induces anoikis in nontransformed cells [17], whereas malignant cells can survive in anchorage-independent culture. MCF-10A, an immortalized human mammary epithelial cell line, forms an acinus-like structure in 3D culture, in which the centrally located cells are removed through anoikis; however, overexpression of HER2/ErbB2 in MCF-10A caused anoikis resistance through the activation of Src-family kinases in 3D culture [44]. Basically, when cells are attached to the scaffold, Src-family kinases are activated by integrins due to the conformational change of Src-family kinases through direct binding on the SH3 domain [15]. The activities of Src-family kinases in mouse embryonic fibroblasts (MEF) was upregulated on cell attachment to the fibronectin scaffold, although this activation was subsequently suppressed by the recruitment of C-terminal Src kinase (Csk) to the membranes harboring activated Src-family kinases [13]. In addition, as observed in ErbB2-expressing MCF-10A cells, many reports showed changes in the activities of Src-family kinases following cell detachment. Connelly et al. showed 20 min of suspension culture activated c-Src in four pancreatic cancer cell lines [45]. Consistently, the activity of c-Src was upregulated in eight lung adenocarcinoma cell lines cultured in suspension for one or two days [46,47]. Lyn, another member of Src-family kinases, was activated in human cervix epithelial HeLa S3 cells within 10 min of cell detachment. Furthermore, Lyn, c-Src, and Fyn were active in suspended HeLa S3 cells for at least two days after cell detachment [48]. However, the kinase activity of c-Src in rat nontransformed small intestinal IEC-18 cells was suppressed by over four hours of suspension culture following transient activation upon cell detachment [49,50]. Wei et al. demonstrated that Src-family kinases were inactive two days following cell detachment in anoikis-sensitive cell lines, Madin–Darby canine kidney (MDCK), human bronchial epithelial, and human airway epithelial (Calu-3) cells, and proposed that activation of Src-family kinases following cell detachment may be linked to anoikis resistance [46]. The different activities of Src-family kinases in suspended cells might be the underlying reason for the difference in anoikis resistance between malignant and non-malignant cells.

Upstream molecules or regulators of Src-family kinases, such as SHP-2 tyrosine phosphatase and platelet-derived growth factor (PDGF) receptor, are involved in the activation of Src-family kinases upon cell detachment [45,46]. However, the mechanisms underlying the transfer of cell detachment signals to the activating Src-family kinases remain to be elucidated. Several mechanisms might be responsible for this observation. First, during dissociation of cells from the surface of culture dishes, the pulling force may activate Src-family kinases. Indeed, application of pulling force on a bead coated with fibronectin and attached on cell surface activated Src-family kinases [51]. Second, changes in membrane curvature might affect the activities of Src-family kinases because changes in the curvature of a particular membrane alter the ratio of the local volume of cytosol or extracellular fluid to the surface area of the membrane, thereby affecting the signaling activity of the receptors [52]. In elliptically cultured cells, the level of active Src-family kinases induced by PDGF treatment was greater in the curved region than in the relatively planar region [53]. Third, because cell detachment can change the pattern of gene expression [40], some of these genes may be involved in the regulation of Src-family kinases.

Loss of cell–scaffold interactions disrupts microtubule polarization, which may interfere with the organization of membrane domains because vectorial trafficking of membranes and proteins along microtubules contributes to apical-basal membrane domain structuring in the plasma membrane [42]. Indeed, cell detachment resulted in the internalization of some lipid raft markers, such as GM1 and caveolin, from the plasma membrane [54,55]. Moreover, cell detachment changed the distribution pattern of c-Src in biochemical fractionation; the distribution of c-Src to the detergent-insoluble fraction was greater in suspended cells than in adherent cells [46]. In our previous report, we have proposed that activation of Src-family kinases in suspended cells is associated with changes in the membrane distributions of these kinases upon cell detachment [48].

## 4. Cell Structure and Cholesterol Distribution

Cholesterol affects the physical properties of biological membranes in many ways. For example, cholesterol limits membrane fluidity, enlarges thickness, and generates intrinsic curvature of lipid bilayers [56]. Similar to changes in the physical forces created by proteins [57], changes in the physical properties resulted from the lipid composition of membranes contribute to generation of the membrane curvature [58]. Moreover, membrane cholesterol affects the mechanical properties of the cytoskeleton: cholesterol depletion strengthens membrane–cytoskeleton adhesion, thereby causing the cell surface membrane to become more rigid [59,60]. Thus, upregulation or downregulation of membrane cholesterol levels can affect the dynamic structure of cellular membranes. Indeed, addition of cholesterol to culture medium changes the structure of red cells from spherical to flat [61]. Cholesterol depletion from the plasma membrane of cells plated on fibronectin results in a round cell structure [62]. Excess levels of cholesterol in human serum (hypercholesterolemia) caused by lipoprotein impairments, such as lecithin-cholesterol acyltransferase deficiency, damage red blood cells and can lead to spur cell anemia [63].

It is interesting to examine whether structural changes in cellular membranes affect the level of membrane cholesterol. Caveolae, 60–80-nm wide cup-shaped structures in the plasma membrane, are cholesterol-rich membrane domains because of direct interaction between cholesterol and caveolin, the core component of caveolae, and undergo endocytosis. Mechanical stress, such as shear stress or cell stretching, causes the caveolae to flatten and merge the components of the caveolar membrane into the plasma membrane [64]. Moreover, like some lipid raft markers (see Section 3), cholesterol in the plasma membrane is internalized upon cell detachment and is recycled back to the plasma membrane after cells are re-plated on fibronectin [48,54]. Structural changes in cellular membranes, therefore, can be a driving force for changes in cholesterol distribution between cellular membranes. It is important to note that the structure of a specific cell cannot solely determine the intracellular distribution of cholesterol. For example, cells undergoing mitosis become spherical in shape; however, the cholesterol distribution in the plasma membrane of mitotic cells is affected by at least the following factors: the ratio of cholesterol distribution between the outer and the inner leaflet of the plasma membrane [65], preferential accumulation of cholesterol in the cleavage furrow [66], and cell-cycle dependent changes in the total cholesterol level per cell [67].

## 5. Changes in the Localizations of Src-Family Kinases upon Cell Detachment

**Figure 1:**
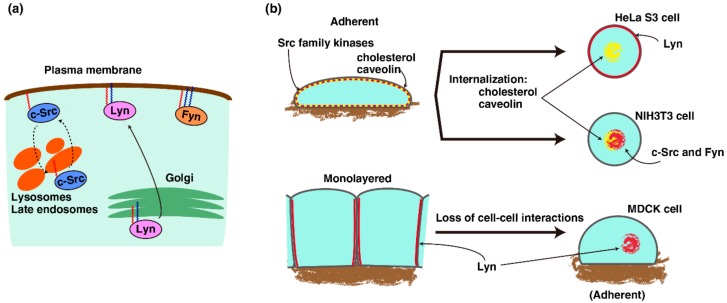
Trafficking of Src-family kinases. (**a**) The black arrow indicates biosynthetic trafficking of Lyn kinase. Distributions of c-Src and Fyn differ from that of Lyn. The dashed arrows indicate the translocation of c-Src between the plasma membrane and endosomes. The red and blue wavy lines represent myristic acids and palmitic acids, respectively; (**b**) Loss of cell–scaffold interactions did not internalize Lyn from the plasma membrane in HeLa S3 cells but internalizes c-Src and Fyn in NIH3T3 cells, whereas caveolin and cholesterol were internalized from the plasma membrane after cell detachment in both cell lines. However, loss of cell–cell interactions is capable of internalizing Lyn in MDCK cells.

Intracellular trafficking of Src-family kinases depends on at least three conditions: (1) biosynthetic trafficking; (2) palmitoylation-dependent distribution; and (3) internalization following cell detachment. First, newly synthesized Lyn and c-Yes initially accumulate in the Golgi region, and are then transported to the plasma membrane (Figure 1a) [11,68,69]. Second, lipid modifications at the N-terminus SH4 domain of Src-family kinases are required to target these kinases to the proper membranes. For example, c-Src is solely myristoylated, whereas Lyn undergoes mono-myristylation and mono-palmitoylation. Unlike Lyn or c-Yes, c-Src, and Lyn (C3S), in which the cysteine residue at position 3 is replaced with a serine residue for the lack of the palmitoylation site, were distributed to the plasma membrane and lysosomes (Figure 1a) [70]. Fyn, another Src-family kinase which undergoes mono-myristylation and di-palmitoylation, is directly targeted to the plasma membrane after protein synthesis [11]. These lipid modifications are important for appropriate functioning of Src-family kinases: palmitoylation is required for accumulation of Lyn to lipid rafts and the involvement of Lyn in lipopolysaccharide-induced signaling in RAW264 macrophage-like cells [71]; palmitoylation of c-Yes mediates oncogenic signaling in HT29 colorectal cancer cells [72]; the lack of lipid modifications in c-Src and Lyn causes chromosome missegregation in mitotic HeLa S3 cells [73]. Third, as with the lipid raft markers that are internalized in suspended cells, Src-family kinases can be translocated from the plasma membrane to endomembranes after loss of cell–cell or cell–scaffold interactions, the pattern of which varies among cell types (Figure 1b). In mouse embryo fibroblast NIH3T3 and MEF cells, c-Src and Fyn, predominantly located at the plasma membrane of the cells in adherent culture, were internalized following the loss of cell–scaffold interactions [55]. Alternatively, in MDCK cells, Lyn and c-Yes, located at the plasma membrane of the cells in monolayer culture, were internalized following the loss of cell–cell interactions [74]. Interestingly, in suspended HeLa S3 cells, in which cell–scaffold and cell–cell interactions were dissociated, Lyn was not internalized from the plasma membrane [48]. Caveolin was located in endomembranes in all the aforementioned cell lines when these cells were in suspended culture. However, while caveolin was colocalized with Src-family kinases in endomembranes in NIH3T3, MEF, and MDCK cells, caveolin in suspended HeLa S3 cells was notably separated from Lyn, which was located at the plasma membrane. Given that caveolin was required for internalization of lipid raft markers and Src-family kinases in suspended MEF cells [55], we hypothesize that nonnegligible changes may occur as a result of the interaction between Lyn and lipid rafts in suspended HeLa S3 cells [67].

## 6. Activation of Lyn Kinase through Cholesterol Depletion after Cell Detachment

### 6.1. Distributions of Src Family Kinases in Membrane Fractions

**Figure 2:**
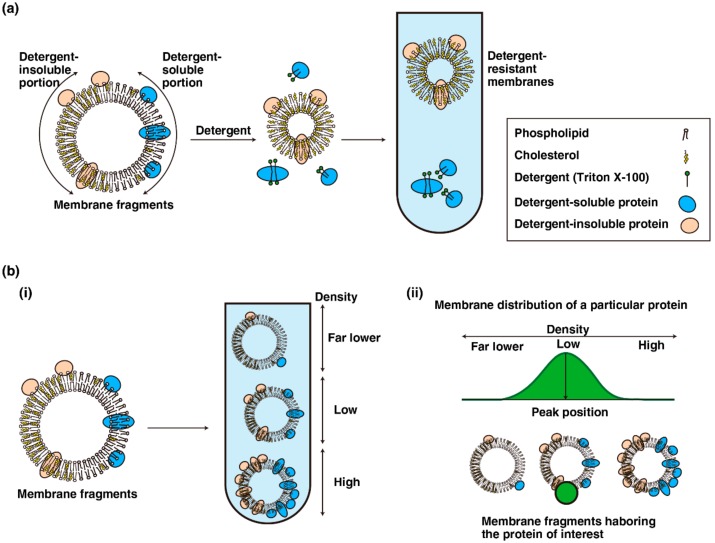
Fractionations of cellular membranes. (**a**) Detergent-based fractionation. An ideal membrane fragment consists of detergent-insoluble portion (left half) and detergent-soluble portion (right half). The proteins in detergent-soluble membranes are solubilized with an appropriate concentration of detergent and separated by density gradient fractionation. (**b**) (**i**) Membrane fragments comprising differential ratios of proteins to lipids were separated by density gradient fractionation; (**ii**) the distribution pattern of a given protein of interest in density gradient fractionation (green area) reflects the density of the membrane segments harboring the protein of interest (green circle).

Detergent-resistant membrane fractionation is a method to isolate detergent-insoluble proteins, which form microsome-like structures in detergent-containing solution that can be separated from detergent-soluble proteins due to their low density [75] (Figure 2a). However, this method is very sensitive to experimental conditions, and the assertion that detergent-resistant membranes indicate the presence of lipid rafts is under debate, as mentioned in Section 2. Detergent-free sucrose-density gradient fractionation is a method to split the bulk of cell-membrane fragments into several fractions based on their density, which varies depending on the ratio of proteins to lipids. The distribution of a particular molecule in this fractionation would reflect the density of each membrane segment harboring that molecule (Figure 2b). We previously used such a detergent-free sucrose-density gradient fractionation method to visualize the membrane distributions of Src-family kinases in adherent and suspension cultures, and found several characteristics as follows. For ease of explanation, we will use three categories to describe membrane fractions: the far lower-density fractions, the low-density fractions, and the high-density fractions. First, the distribution pattern of Lyn is different from that of c-Src in adherent cells [48,70,74,76]. Lyn is primarily distributed in the low-density fractions, whereas c-Src is distributed in the far lower-density fractions in adherent HeLa S3, parental HeLa, and human embryonic kidney HEK293 cells. The distribution of c-Src in monolayered MDCK cells was slightly different from that in the other aforementioned cell lines: the majority of c-Src was spread from the far lower-density fractions to the low-density fractions [74]. The difference between the distributions of c-Src and Lyn may be associated with the palmitoylation status of Src-family kinases because the distribution pattern of the Lyn(C3S) mutant was very similar to that of c-Src [48,74]; moreover, other palmitoylated members of Src-family kinases, c-Yes and Fyn, were distributed to the low-density fractions like Lyn [48].

Second, cell detachment affected the membrane distributions of Src-family kinases. In HeLa S3 cells, the main peaks of the distributions of Lyn, c-Yes and Fyn shifted from the low-density fractions to the high-density fractions upon cell detachment, whereas each distribution pattern of representative membrane proteins—i.e., desmoglein, galactosyltransferase, transferrin receptor, and calnexin—were largely unchanged by cell detachment [48]. Interestingly, the majority of c-Src in HeLa S3 cells was distributed in the far lower-density fractions in both adherent and suspension cultures, whereas in MDCK cells, the distribution of c-Src was accumulated from the low-density fractions to the far lower-density fractions by cell detachment, although Lyn distribution remain largely unchanged upon cell detachment. The reasons for these differences in the distributions of Src-family kinases between HeLa S3 and MDCK cells are still unclear, but variations in the localization of Lyn may play a role in determining the distribution of Lyn in these cell lines. As we discussed in Section 3 through Section 5, cell detachment translocates caveolin, cholesterol, and lipid raft-related molecules from the plasma membrane; however, Lyn remained localized at the plasma membrane in suspended HeLa S3 cells. Caveolae accumulate many types of signal-transduction proteins, including Src-family kinases [77], and have a unique density [78]; and cholesterol can affect physical properties of cellular membranes (see Section 2). We treated suspended HeLa S3 cells with an inhibitor for dynamin-2, which regulates membrane fission of caveolar vesicles and MβCD-cholesterol, which replenishes cholesterol at the plasma membrane. As a result of this treatment, Lyn and Fyn were distributed to the low-density fraction in suspended HeLa S3 cells [48].

### 6.2. Role of the Changes in the Lyn Distribution in Lyn Activation upon Cell Detachment

Analyses examining the relationship between the membrane distributions and the activities of Src-family kinases have been conducted using these methods to isolate detergent-resistant membranes. Some reports demonstrated that Src-family kinases are active in detergent-resistant membranes [79,80], whereas others demonstrated that Src-family kinases can be inactivated in detergent-resistant membranes as a result of Csk recruitment along with its adaptor molecule Csk-binding protein (Cbp) [81]. Moreover, some detergent-free fractionation methods have been developed to isolate particular membrane domains [78,82]. In our fractionation experiment, the activity of Lyn present in the high-density fractions was greater than that in the low-density fraction in adherent HeLa S3 cells, although the population of Lyn present in the high-density fraction was smaller than that in the low-density fractions when cells were cultured in adherent conditions. Moreover, cholesterol replenishment, not dynamin inhibition, attenuated the activation of Lyn in suspension culture, whereas cholesterol depletion followed by serum stimulation activated Lyn in adherent culture [48]. Regulation of the activities of Src-family kinases involves many proteins, such as Csk, SHP-2, and EGFR [36,45,81]. The membrane distributions of Csk and SHP-2 were limited to the far lower-density fractions in both adherent and suspension cultures, whereas that of EGFR was shifted from the low-density fractions to the high-density fractions upon cell detachment, like Lyn distribution. Moreover, Lyn and EGFR were colocalized at the plasma membrane in suspended HeLa S3 cells. EGFR can be activated by cholesterol depletion (see Section 2), even though cholesterol-rich membranes are also considered to play a role in EGFR activation [83]. These facts collectively suggest that cell detachment reduces cholesterol levels in membrane segments harboring Lyn and EGFR to augment the activation of Lyn by EGFR signaling.

## 7. Conclusions and Perspectives

**Figure 3:**
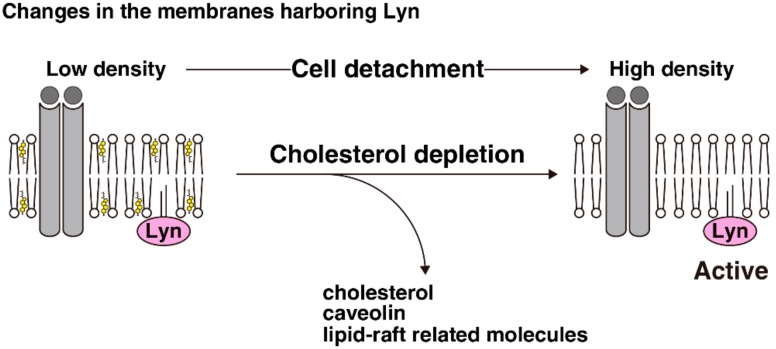
Cell detachment activates Lyn through cholesterol depletion. Cell detachment causes internalization of some lipid raft-related molecules, including caveolin and cholesterol, in the plasma membrane. Cholesterol depletion affects the characteristics of the membrane segments harboring Lyn, which is associated with activation of Lyn in suspended cells.

Cholesterol is heterogeneously distributed between membranes and forms cholesterol-rich membrane raft domains, and may recruit particular molecules—such as GM1 and caveolin—and interfere with signal transduction at cellular membranes. Loss of cell–scaffold interactions translocates cholesterol and some lipid raft-related molecules from the plasma membrane to endomembranes; moreover, Src-family kinases, which can be distributed to both lipid rafts and non-raft membranes, are primarily localized to either endomembranes or the plasma membrane depending on cell lines in suspension culture. Cell detachment notably alters the patterns of membrane distributions of Src-family kinases, particularly Lyn and Fyn, in detergent-free density gradient fractionation in the cells in which cell detachment splits the main localizations of Src-family kinases and cholesterol between the plasma membrane and endomembranes. Following cholesterol enrichment and dynamin-2 inhibition, the membrane distributions of Lyn and Fyn in suspended cells becomes similar to that in adherent cells. This suggests that cholesterol internalization upon cell detachment might cause membrane cholesterol depletion with subsequent physical changes in the characteristics of the membrane segments harboring Src-family kinases. Deprivation or enrichment of cellular cholesterol can change the activities of some signaling molecules at the plasma membrane; cholesterol deprivation activates Lyn in adherent cells, whereas cholesterol enrichment attenuates Lyn activation in suspended cells. Therefore, membrane cholesterol depletion may play a role in the activation of Src-family kinases following cell detachment (Figure 3). Activation of Src-family kinases in suspended cells may be involved in anoikis resistance [44,45,46,47,48], one of the features of cancer malignancy, whereas the relationship between serum cholesterol level and cancer mortality remains controversial [2]. Different cell types differentially regulate the localizations of Src-family kinases, and variations in the cholesterol levels of the membrane segments to which these kinases are localized may influence their activities. The effects of membrane cholesterol levels on the activities of Src-family kinases may be associated with anoikis resistance in malignant cancer cells.

## Abbreviations

Cbp	Csk-binding protein
Csk	C-terminal Src kinase
ECM	Extracellular matrix
EGFR	Epidermal growth factor receptor
ER	Endoplasmic reticulum
LDL	Low-density lipoprotein
MβCD	Methyl-β-cyclodextrin
MEF	Mouse embryonic fibroblast
MDCK	Madin–Darby canine kidney
NPC	Niemann–Pick disease type C
PDGF	Platelet-derived growth factor
SH	Src homology
